# Urbanization, economic development and health: evidence from China’s labor-force dynamic survey

**DOI:** 10.1186/s12939-017-0705-9

**Published:** 2017-11-29

**Authors:** Hongsheng Chen, Ye Liu, Zhigang Li, Desheng Xue

**Affiliations:** 1 School of Geography and Planning, Sun Yat-Sen University, Xingang Xi Road, Guangzhou, 510275 China; 2 School of Architecture, Southeast University, Si-Pai-Lou Road No.2, Nanjing, 210096 China; 3 School of Urban Design, Wuhan University, Ba-Yi Road No.299, Wuhan, 430072 China

**Keywords:** Urbanization, Economic development, Health, Income inequality, China

## Abstract

**Background:**

The frequent outbreak of environmental threats in China has resulted in increased criticism regarding the health effects of China’s urbanization. Urbanization is a double-edged sword with regard to health in China. Although great efforts have been made to investigate the mechanisms through which urbanization influences health, the effect of both economic development and urbanization on health in China is still unclear, and how urbanization-health (or development-health) relationships vary among different income groups remain poorly understood. To bridge these gaps, the present study investigates the impact of both urbanization and economic development on individuals’ self-rated health and its underlying mechanisms in China.

**Methods:**

We use data from the national scale of the 2014 China Labor-force Dynamics Survey to analyze the impact of China’s urbanization and economic development on health. A total of 14,791 individuals were sampled from 401 neighborhoods within 124 prefecture-level cities. Multilevel ordered logistic models were applied.

**Results:**

Model results showed an inverted U-shaped relationship between individuals’ self-rated health and urbanization rates (with a turning point of urbanization rate at 42.0%) and a positive linear relationship between their self-rated health and economic development. Model results also suggested that the urbanization-health relationship was inverted U-shaped for high- and middle-income people (with a turning point of urbanization rate at 0.0% and 49.2%, respectively), and the development-health relationship was inverted U-shaped for high- and low-income people (with turning points of GDP per capita at 93,462 yuan and 71,333 yuan, respectively) and linear for middle-income people.

**Conclusion:**

The impact of urbanization and economic development on health in China is complicated. Careful assessments are needed to understand the health impact of China’s rapid urbanization. Social and environmental problems arising from rapid urbanization and economic growth should be addressed. Equitable provision of health services are needed to improve low-income groups’ health in highly urbanized cities.

## Background

The growing body of evidence suggests that individual health is closely related to urbanization [1–4]. China is experiencing urbanization at an unparalleled pace, presenting opportunities to investigate the effect of urbanization on health in developing countries [5, 6]. Urbanization is a double-edged sword with regard to Chinese people’s health [6–8]. On the one hand, it may improve health benefits: urban populations tend to have better access to health services and health knowledge than rural populations do. On the other hand, it may increase Chinese people’s exposure to risk factors for chronic non-communicable diseases such as air pollution, unhealthy diets, sedentary lifestyles, and life stresses.

The body of literature on urbanization-health relationships in China can be categorized into those that investigate the direct relationship between urbanization rates and residents’ health [2, 9, 10] and those that examine the effect of environmental hazards arising from urbanization (e.g. increased air pollution) on residents’ health [11–13]. For example, using longitudinal data from the China Health and Nutrition Survey, van de Poel et al. [2] found that urbanization raised the probability of reporting poor health. Chen et al.’s [10] study showed that rapid and accelerated urbanization improved overall health. Miao and Wu [14] found that living in more-advanced communities increased the risk of chronic disease in China and suggested that unhealthy lifestyles was a pathway through which urbanization influenced individuals’ health. Although great efforts have been made to investigate the mechanisms through which urbanization influences health, the effect of both economic development and urbanization on health in China is still unclear, and how urbanization-health (or development-health) relationships vary among different income groups remain poorly understood.

To bridge these gaps, the present study investigates the impact of both urbanization and economic development on individuals’ self-rated health and its underlying mechanisms in China using data from the 2014 wave of China Labor-force Dynamics Survey (CLDS 2014). We further examine how the effects of urbanization and economic development vary among different income groups. This study contributes to the body of literature in two respects: first, it enhances our understanding of factors shaping people’s self-rated health in China by considering the effects of both urbanization and economic development; second, it provides a deeper understanding of urbanization-development-health relationships by examining variance across income groups.

## Methods

### Data

This study primarily used data from the CLDS 2014 conducted by the Center for Social Science Survey of Sun Yat-sen University in collaboration with 27 higher education institutions in China in 2013–2014 [15]. The survey team selected respondents using a multistage, cluster, stratified, Probability-Proportional-to-Size (PPS) sampling technique. In the first stage of the survey, 124 prefecture-level divisions were chosen from 29 provinces across China. The second stage of the survey involved a random selection of 401 neighborhoods from the sampled divisions. In the context of China, prefectures refer to the second-level administrative divisions (including prefectures, prefecture-level cities and leagues), and neighborhoods refer to the fifth-level administrative divisions (including neighborhoods in urban areas and villages in rural areas). A neighborhood is nested within a prefecture. GDP per capita, population size, and urbanization-rate data were gathered from the China City Statistical Yearbook. After excluding observations with missing essential information, the final dataset contained 14,791 individuals nested within 401 neighborhoods nested within 124 prefecture-level cities.

### Methods

We examined the effects of regional variation in urbanization and economic development on respondents’ self-rated health, using three-level ordered logistic regressions. Multilevel models were particularly suitable for this research design, as the CLDS 2014 data have a hierarchical structure with individuals nested within neighborhoods which are nested within cities. Table 1 presents the summary statistics of variables in the regression models. The dependent variable is respondents’ self-rated health, assessed by the following question in CLDS questionnaires: “how would you rate your current health status according to the following five categories, very good, good, fair, poor, or very poor?” Overall, 10.60% of the sample reported poor health (very poor and poor), 63.72% reported good health (very good and good) and 25.68% reported that their health was neither good nor bad. Furthermore, to examine how urbanization-health relationships vary by respondents’ income, we divided the respondents into three categories: low-income group (annual personal income less than 10,000 yuan), middle-income group (annual personal income ranging between 10,000 yuan and 30,000 yuan), and high-income group (annual personal income more than 30,000 yuan). Table 2 shows the cross-tabulation of income groups by self-rated health. The percentage of respondents of high-income group reporting good health (74.57%) is larger than those of low- and middle-income group (46.42% and 63.35%, respectively). The results showed that higher-income people were more likely to report good health than low-income people.

**Table 1:** Summary statistics of variables included in regressions

Dependent variables	
Self-rated health (%)	Proportion/Mean (SD)
Very poor	1.01
Poor	9.59
Fair	25.68
Good	41.92
Very good	21.80
Independent variables (prefecture-level variables)	
Urbanization rate in 2014 (%)	56.23 (18.10)
GDP per capita in 2014 (10,000 yuan)	5.55 (3.18)
The change of urbanization rate from 2010 to 2014 (%)	4.28 (2.75)
The population size (million people)	6.20 (4.69)
Control variables (demographic and socioeconomic variables)	
Age	44.57 (12.67)
Gender (%)	
Female	44.03
Male	55.97
Marital status (%)	
Single, divorced, or widowed	12.98
Married	87.02
Education (%)	
Primary school and below	35.22
Junior high school	33.47
Senior high school	16.47
College and above	14.84
Employment (%)	
Unemployed	6.19
Employed	93.81
Annual personal income (Yuan)	32,729.64 (85,361.41)
*Hukou* status (%)	
Non-local *hukou*	18.59
Local *hukou*	81.41
Length of residence in the city (year)	39.70 (17.20)
Neighborhood (%)	
Urban neighborhood	35.74
Rural neighborhood	64.26
Smoking (%)	
Smoker	29.87
Former smoker	3.56
Nonsmoker	66.57
Physical exercise (%)	
Frequent	18.38
Infrequent	81.62

**Table 2:** Tabulation between respondents’ income groups and their self-rated health

Self-rated health		Low-income group (<10,000 yuan)	Middle-income group (10,000–30,000 yuan)	High-income group (>30,000 yuan)	Total
Very poor	Sample size	89	47	14	150
	Proportion (%)	2.51	0.87	0.24	1.01
Poor	Sample size	717	463	238	1418
	Proportion (%)	20.24	8.53	4.09	9.59
Fair	Sample size	1092	1479	1228	3799
	Proportion (%)	30.83	27.25	21.10	25.68
Good	Sample size	1224	2281	2694	6199
	Proportion (%)	34.56	42.02	46.28	41.91
Very good	Sample size	420	1158	1647	3225
	Proportion (%)	11.86	21.33	28.29	21.81
Total		3542 (23.94%)	5428 (36.70%)	5821 (39.36%)	14,791
χ^2^		1200			
*p*-value		0.000			

Two prefecture-level variables were used as independent variables in the regression models: urbanization rates and GDP per capita. We used a prefecture-level ratio of urban to total population to measure the city’s urbanization rate. In keeping with previous studies on regional inequality in China [16, 17], we used GDP per capita of the prefecture-level cities to measure the level of economic development. Given that the relationship between individuals’ self-rated health and the level of urbanization (or economic development) may be U-shaped curvilinear rather than linear, we considered the squares of urbanization rate and GDP per capita in the regression models.

We included two prefecture-level controlled variables, the change of urbanization rate over the period of 2010–2014 and the logarithm of population size, in the regressions. In addition, we controlled a series of individual-level variables including age (continuous variable), gender (dichotomous variable), marital status (dichotomous variable), educational attainment (categorical variables), employment status (dichotomous variable), annual personal income (continuous variable), *hukou* status (household registration status, dichotomous variable), length of residence in the city (continuous variable), neighborhood type (urban or rural, dichotomous variable), smoking history (categorical variables), and the frequency of physical exercise (dichotomous variable).

## Results

Table 3 shows the results of the multilevel ordered logistic models on respondents’ self-rated health. Model 1 included individual-level control variables only. The odds of respondents reporting good health decreased with age (OR = 0.963, 95% CI: 0.959–0.967). Compared with women, men were more likely to report good health (OR = 1.162, 95% CI: 1.073–1.258). Respondents with higher educational attainment were more likely to report good health than those who had primary school education or below (junior high school, OR = 1.276, 95% CI: 1.173–1.387; senior high school, OR = 1.357, 95% CI: 1.217–1.512; college and above, OR = 1.438, 95% CI: 1.260–1.642). Employed respondents were more likely to report good health than unemployed respondents (OR = 1.196, 95% CI: 1.049–1.364). The odds of reporting good health increased with the logarithm of annual personal income (OR = 1.235, 95% CI: 1.194–1.278). Former smokers reported poorer health than those who were smokers (OR = 0.758, 95% CI: 0.636–0.902). This may be attributed to the tendency of former smokers to quit smoking after their health worsened. Further, respondents who frequently performed physical exercise were more likely to report good health than those who performed exercise infrequently (OR = 1.141, 95% CI: 1.046–1.245).

**Table 3:** Multilevel mix-effect ordered logistic regression estimates

	Model 1		Model 2		Model 3	
	OR	95% CI	OR	95% CI	OR	95% CI
Independent variables						
Urbanization rate			0.992	[0.980, 1.004]	1.043*	[0.995, 1.092]
Square of urbanization rate					1.000**	[0.999, 1.000]
GDP per capita in 2014			1.057	[0.983, 1.137]	1.250**	[1.022, 1.529]
Square of GDP per capita in 2014					0.989	[0.976, 1.003]
Control variables						
The change of urbanization rate from 2010 to 2014			0.998	[0.948, 1.051]	0.986	[0.935, 1.041]
The logarithm of population size of the prefecture-level city			1.130	[0.935, 1.366]	1.150	[0.962, 1.374]
Age	0.963***	[0.959, 0.967]	0.963***	[0.959, 0.967]	0.963***	[0.959, 0.967]
Male (ref: female)	1.162***	[1.073, 1.258]	1.163***	[1.074, 1.259]	1.165***	[1.075, 1.261]
Married (ref: single- divorced- or widowed)	0.927	[0.838, 1.027]	0.928	[0.839, 1.028]	0.928	[0.838, 1.028]
Education (ref: primary school or below)						
Junior high school	1.276***	[1.173, 1.387]	1.275***	[1.173, 1.387]	1.273***	[1.171, 1.384]
Senior high school	1.357***	[1.217, 1.512]	1.356***	[1.217, 1.512]	1.351***	[1.212, 1.506]
College and above	1.438***	[1.260, 1.642]	1.438***	[1.260, 1.641]	1.430***	[1.253, 1.632]
Employed (ref: unemployed)	1.196***	[1.049, 1.364]	1.196***	[1.049, 1.363]	1.197***	[1.050, 1.365]
Logarithm of annual personal income	1.235***	[1.194, 1.278]	1.234***	[1.192, 1.276]	1.233***	[1.191, 1.275]
Local *hukou* (ref: non-local *hukou*)	1.030	[0.935, 1.135]	1.031	[0.936, 1.136]	1.025	[0.931, 1.130]
Length of residence in the city	1.002	[0.999, 1.006]	1.002	[0.999, 1.006]	1.002	[0.999, 1.006]
Urban neighborhood (ref: rural neighborhood)	1.111	[0.943, 1.309]	1.092	[0.925, 1.290]	1.093	[0.927, 1.288]
Smoking (ref: smoker)						
Former smoker	0.758***	[0.636, 0.902]	0.757***	[0.636, 0.902]	0.757***	[0.636, 0.901]
Nonsmoker	0.947	[0.870, 1.031]	0.947	[0.870, 1.031]	0.947	[0.870, 1.031]
Frequent physical exercises (ref: infrequent physical exercises)	1.141***	[1.046, 1.245]	1.142***	[1.047, 1.246]	1.144***	[1.048, 1.248]
Number of individuals	14,791		14,791		14,791	
Number of neighborhoods	401		401		401	
Number of cities	124		124		124	
Log likelihood	−17,813.211		−17,811.009		−17,802.663	
χ^2^	1321.023***		1325.168***		1345.478***	

*OR* odds ratio, *CI* confidence interval. *p < 0.10, **p < 0.05, ***p < 0.01

Models 2 included not only individual-level variables but also prefecture-level variables in their linear form. Interestingly, there is no evidence that a significant linear relationship between urbanization rates (or GDP per capita) and respondents’ self-rated health exists. Although independent variables in Model 2 were not significant, when their quadratic forms were added in Model 3, both of their quadratic form and linear form became statistically significant (urbanization rate: *p* < 0.10; square of urbanization rate: *p* < 0.05; GDP per capita in 2014: *P* < 0.05). Model results show a significant inverted U-shaped relationship between the odds of reporting good health and urbanization rate. Specifically, a respondent would have the highest odds of reporting good health if he or she lives in a region where its urbanization rate is around 42.0% when other variables are controlled (urbanization rate: OR = 1.043, 90% CI: 0.995–1.092; square of urbanization rate: OR = 1.000, 95% CI: 0.999–1.000).

We performed separate multilevel ordered logistic regressions for high-, middle- and low-income respondents (Table 4). In terms of the relationship between urbanization and health, Model 9 shows an inverted U-shaped relationship between urbanization and individuals’ self-rated health for high-income group. A high-income individual would have the highest odds of reporting good health if he or she lives in a region where its urbanization rate is around 49.2% when other variables are controlled (urbanization rate: OR = 1.061, 95% CI: 1.004–1.121; square of urbanization rate: OR = 0.999, 99% CI: 0.999–1.000). Model 7 suggests that the odds of middle-income people reporting good health decreases with increased urbanization rate (square of urbanization rate: OR = 1.000, 90% CI: 0.999–1.000). However, no evidence suggests that low-income people’s self-rated health is significantly associated with urbanization level (Models 4 and 5).

**Table 4:** Multilevel mix-effect ordered logistic regression estimates, by income group

	Low-income group				Middle-income group				High-income group			
	Model 4		Model 5		Model 6		Model 7		Model 8		Model 9	
	OR	95% CI	OR	95% CI	OR	95% CI	OR	95% CI	OR	95% CI	OR	95% CI
Independent variables												
Urbanization rate	1.001	[0.987, 1.015]	1.004	[0.953, 1.058]	0.991	[0.978, 1.005]	1.039	[0.984, 1.096]	0.991	[0.977, 1.004]	1.061**	[1.004, 1.121]
Square of urbanization rate			1.000	[0.999, 1.000]			1.000*	[0.999, 1.000]			0.999***	[0.999, 1.000]
GDP per capita in 2014	1.013	[0.929, 1.104]	1.238*	[0.983, 1.559]	1.081*	[0.996, 1.174]	1.298**	[1.031, 1.635]	1.048	[0.967, 1.137]	1.275**	[1.017, 1.598]
Square of GDP per capita in 2014			0.986*	[0.969, 1.002]			0.988	[0.973, 1.004]			0.988*	[0.973, 1.002]
Control variables												
The change of urbanization rate from 2010 to 2014	0.999	[0.945, 1.056]	1.003	[0.945, 1.064]	1.026	[0.967, 1.088]	1.017	[0.957, 1.081]	0.979	[0.921, 1.040]	0.966	[0.909, 1.027]
The logarithm of population size of the prefecture-level city	1.136	[0.918, 1.404]	1.164	[0.943, 1.436]	1.171	[0.947, 1.448]	1.189*	[0.971, 1.457]	1.127	[0.908, 1.401]	1.127	[0.923, 1.375]
Age	0.960***	[0.951, 0.970]	0.960***	[0.951, 0.970]	0.961***	[0.954, 0.969]	0.961***	[0.954, 0.968]	0.969***	[0.962, 0.976]	0.969***	[0.962, 0.976]
Male (ref: female)	1.174*	[0.987, 1.397]	1.176*	[0.988, 1.398]	1.261***	[1.100, 1.445]	1.266***	[1.105, 1.452]	1.086	[0.957, 1.233]	1.086	[0.956, 1.232]
Married (ref: single- divorced- or widowed)	1.018	[0.832, 1.246]	1.019	[0.833, 1.247]	0.910	[0.764, 1.085]	0.910	[0.764, 1.085]	0.852*	[0.722, 1.005]	0.852*	[0.722, 1.005]
Education (ref: primary school or below)												
Junior high school	1.260***	[1.075, 1.478]	1.258***	[1.073, 1.476]	1.226***	[1.078, 1.396]	1.221***	[1.073, 1.390]	1.306***	[1.109, 1.539]	1.301***	[1.104, 1.532]
Senior high school	1.156	[0.893, 1.496]	1.148	[0.886, 1.486]	1.355***	[1.141, 1.609]	1.344***	[1.132, 1.596]	1.351***	[1.121, 1.629]	1.339***	[1.111, 1.614]
College and above	1.596*	[0.984, 2.589]	1.555*	[0.958, 2.524]	1.741***	[1.357, 2.233]	1.715***	[1.338, 2.200]	1.396***	[1.141, 1.707]	1.377***	[1.127, 1.684]
Employed (ref: unemployed)	1.137	[0.885, 1.462]	1.135	[0.883, 1.458]	1.234**	[1.012, 1.505]	1.239**	[1.016, 1.511]	1.245*	[0.960, 1.615]	1.253*	[0.966, 1.625]
Logarithm of annual personal income	1.107**	[1.015, 1.208]	1.102**	[1.010, 1.202]	1.212**	[1.026, 1.431]	1.203**	[1.019, 1.420]	1.085*	[0.985, 1.196]	1.087*	[0.986, 1.198]
Local *hukou* (ref: non-local *hukou*)	1.021	[0.755, 1.382]	0.988	[0.729, 1.341]	1.068	[0.903, 1.263]	1.059	[0.895, 1.252]	1.019	[0.892, 1.165]	1.006	[0.881, 1.150]
Length of residence in the city	1.006*	[0.999, 1.014]	1.006	[0.999, 1.014]	1.002	[0.996, 1.007]	1.001	[0.996, 1.007]	1.002	[0.997, 1.007]	1.002	[0.997, 1.007]
Urban neighborhood (ref: rural neighborhood)	1.285	[0.948, 1.741]	1.322*	[0.976, 1.792]	1.022	[0.827, 1.262]	1.027	[0.834, 1.266]	0.938	[0.755, 1.166]	0.932	[0.7531.154]
Smoking (ref: smoker)												
Former smoker	0.883	[0.612, 1.273]	0.886	[0.614, 1.279]	0.843	[0.621, 1.144]	0.841	[0.619, 1.141]	0.621***	[0.476, 0.811]	0.619***	[0.475, 0.808]
Nonsmoker	1.063	[0.878, 1.288]	1.062	[0.877, 1.286]	0.969	[0.837, 1.123]	0.970	[0.838, 1.124]	0.912	[0.802, 1.037]	0.910	[0.800, 1.035]
Frequent physical exercises (ref: infrequent physical exercises)	1.052	[0.856, 1.294]	1.055	[0.858, 1.297]	1.122	[0.960, 1.311]	1.124	[0.962, 1.314]	1.213***	[1.070, 1.375]	1.216***	[1.073, 1.378]
Number of individuals	3542		3542		5428		5428		5821		5821	
Number of neighborhoods	335		335		394		394		394		394	
Number of cities	124		124		124		124		124		124	
Log likelihood	−4715.645		−4712.972		−6584.833		−6578.379		−6542.014		−6532.229	
χ^2^	251.451***		256.922***		408.766***		421.789***		235.102***		254.813***	

*OR* odds ratio, *CI* confidence interval. *p < 0.10, **p < 0.05, ***p < 0.01

Regarding the effect of economic development, Model 3 shows a linear development-health relationship for all respondents (GDP per capita: OR = 1.250, 95% CI: 1.022–1.529). When regressions were run separately for different income groups, an inverted U-shaped relationship was observed for the low- and high-income group, whereas a linear relationship was observed for the middle-income group. Specifically, the odds of a high-income individual reporting good health reached its peak when he or she lived in a city where GDP per capita in 2014 was around 93,462 yuan (GDP per capita: OR = 1.275, 95% CI: 1.017–1.598; square of GDP per capita: OR = 0.988, 90% CI: 0.973–1.002). With respect to low-income individuals, the odds reached its peak where GDP per capita was around 71,333 yuan (GDP per capita: OR = 1.238, 90% CI: 0.983–1.559; square of GDP per capita: OR = 0.986, 90% CI: 0.969–1.002).

## Discussion

Contradicting previous research [2, 10], the present study found that the urbanization-health and/or development-health relationships were inverted U-shaped curvilinear rather than linear. China has undergone rapid urbanization over the past three decades, where two main trends have been observed: the migration of people from rural to urban areas for economic purposes [18, 19] and the in-situ urbanization through which residents of rural areas are granted urban citizenship and welfare at the expense of ownership of farmland and housing land [20]. Urbanization is usually accompanied by the improvement of health services and distribution of health-related knowledge [21, 22]. In this sense, urbanization helps promote people’ health. However, urban population are more likely than rural population to be exposed to several health risks such as environmental pollution, unhealthy lifestyles, and life stresses. In addition, migrants—who comprise a significant proportion of the population in large cities—suffer from poor living conditions, inadequate health services, and high work pressures. These detrimental environmental and occupational factors exert a negative influence on their health [23, 24]. Consequently, urbanization is a double-edged sword with regard to health in China.

The relationship between the level of economic development and residents’ self-rated health also formed an inverted U-shaped curve. During the planned-economy period, China’s economic development was lower than the global average and the standard of living was very low. After the opening of the market, China achieved rapid economic growth, becoming the world’s second largest economy in 2010 [25, 26]; this improved general living standards in China [27]. Furthermore, health services in economically developed cities are better than those in less economically developed cities. Unsurprisingly, improved economic development had a positive effect on residents’ health. Although rapid economic development may result in environmental and social problems such as serious air pollution and social inequality [28, 29], no evidence suggests that the positive association between economic development and individuals’ health status changes with the level of economic development.

The impact of urbanization and economic development on health varied across high-, middle- and low-income groups. Consistent with previous studies [30, 31], we found that the higher residents’ income was, the more likely they were to report good health, and vice versa. Moreover, economic development were significantly linked to low-income residents’ self-rated health (inverted U-shaped relationship). This phenomena is similar to the “metropolitan involution” [32]. That is, the benefits brought by the metropolization and urbanization will reach the limit for low-income group at certain stage of economic development. After that, metropolization and urbanization do not necessarily break the structural inequalities (social, economic and regional) and also produce new types of inequalities.

In China, low-income group is more vulnerable to detrimental urban social and physical environments than high-income group [1]. China’s social welfare for low-income groups needs improvement, especially since most low-income families are unable to afford commercial insurance and are therefore vulnerable to health problems. Additionally, even in economically developed cities with a higher rate of health services, low-income group tended to have limited access to high-quality health services. In China, the quality of health services varies greatly among different cities and regions. Generally speaking, health services in big cities are superior to health services in small and medium-sized cities, and urban health services are superior to those in rural areas. Moreover, China’s high-quality health services are not universal. On the contrary, they are available only for those who are able to afford them. Therefore, the urbanization and urban modernization in China does not necessarily lead to the improvement of all residents’ health, because low-income people, especially low-income migrant workers, may have little access to good health services in large cities due to their limited financial capability.

China’s rapid urbanization and economic growth in the past 30 years are associated with increased health risks, including environmental pollution, unbalanced provision of medical care, and sedentary lifestyles. Nowadays, Chinese local governments are keen to pursue a higher urbanization rate and a higher economic growth rate. However, they seldom take into consideration the negative health effects of urbanization and economic growth. Our study suggests that regions/ cities with a high urbanization rate are not necessarily places most conducive to residents’ health. On the contrary, regions/ cities with a medium urbanization rate are more conducive than other regions/cities to the health of their residents. Therefore, Chinese local governments are advised to pay more attention to the quality rather than the pace of urbanization and take actions to curb the negative impact of urbanization on health. Simultaneously, the Chinese government needs to strengthen environmental regulation, reduce environmental pollution, and improve rural and urban standards of living. Compared to high-income people, the health status of low-income people is more likely to rapidly decrease with the rapid growth of the city’s economy. Therefore, we propose that low-income people deserve more health benefits than high-income people. To decrease health disparity between income groups, more public resources should be allocated to provide more affordable high-quality health services to low-income people. Moreover, policies should address the problem of regional disparity in economic development. The Chinese central government should also increase investment in basic public services in poorer areas to improve residents’ living conditions and environment.

Despite the study’s contributions, some research limitations should be noted. First, we were unable to investigate the causal effect of changes in urbanization and economic development over time on individuals’ health outcomes due to the cross-sectional nature of the data. Second, we did not investigate pathways (e.g., lifestyles) through which urbanization and economic development influence residents’ health. For instance, some scholars have found that residents of highly urbanized areas tend to engage in fewer physical activities, have a higher calorie intake, and smoke more frequently, thereby increasing their health risks [2, 33]. Third, the measurement of health is based on respondents’ self-rating. It should be noted that there may be a gap between respondents’ self-rated health status and the objective conditions of their health status.

## Conclusion

This study contributes to the understanding of the health impact of urbanization and economic development in China. The three-level ordered logistic regressions showed an inverted U-shaped relationship between individuals’ self-rated health and urbanization rates and a positive linear relationship between their self-rated health and economic development. When the prefecture-level city’s urbanization rate was less than 42.0%, respondents’ self-rated health increased with increased urbanization rates. However, when the urbanization rate was higher than 42.0%, the level of respondents’ self-rated health decreased with increased urbanization rates. Model results also suggested that the urbanization-health relationship was inverted U-shaped for high- and middle-income people (with a turning point of urbanization rate at 0.0% and 49.2%, respectively), and the development-health relationship was inverted U-shaped for high- and low-income people (with turning points of GDP per capita at 93,462 yuan and 71,333 yuan, respectively) and linear for middle-income people.

## Abbreviations

CI: Confidence interval
CLDS: China labor-force dynamics survey
GDP: Gross domestic product
OR: Odds ratio
PPS: Probability-proportional-to-size
